# Benchmark for Time in Therapeutic Range in Venous Thromboembolism: A Systematic Review and Meta-Analysis

**DOI:** 10.1371/journal.pone.0042269

**Published:** 2012-09-25

**Authors:** Petra M. G. Erkens, Hugo ten Cate, Harry R. Büller, Martin H. Prins

**Affiliations:** 1 Department of Family Medicine, School for Public Health and Primary Care and Cardiovascular Research Institute Maastricht, Maastricht University Medical Centre, Maastricht, The Netherlands; 2 Department of Internal Medicine and Cardiovascular Research Institute Maastricht, Maastricht University Medical Centre, Maastricht, The Netherlands; 3 Department of Vascular Medicine, Academic Medical Center, Amsterdam, The Netherlands; 4 Department of Clinical Epidemiology and Medical Technology Assessment, Maastricht University Medical Centre, Maastricht, The Netherlands; Institut National de la Santé et de la Recherche Médicale, France

## Abstract

**Introduction:**

The percentage of time within the target INR range 2.0 to 3.0 (TTR) in patients treated with vitamin K antagonists varies considerably among efficacy-studies of novel anticoagulants. In order to properly asses the quality of anticoagulant control in upcoming cost-effectiveness studies and real life registries this systematic review reports a benchmark of TTR for different treatment durations in patients with venous thromboembolism and discusses ways to calculate TTR.

**Methods:**

Medline and Embase were searched for studies published between January 1990 and May 2012. Randomized controlled trials and cohort studies reporting the TTR in patients with objectively confirmed venous thromboembolism treated with vitamin K antagonists (VKA) were eligible. Duplicate reports, studies only reporting INR during initial treatment or with VKA treatment less than 3 months were excluded. Three authors assessed trials for inclusion and extracted data independently. Discrepancies were resolved by discussion between the reviewers. A meta-analysis was performed by calculating a weighted mean, based on the number of participants in each included study, for each time-period in which the TTR was measured since the confirmation of the diagnosis of VTE.

**Results:**

Forty studies were included (26064 patients). The weighted means of TTR were 54.0% in the first month since the start of treatment, 55.6% in months 1 to 3, 60.0% in months 2 to 3, 60.0% in the months1 to 6+ and 75.2% in months 4 to 12+. Five studies reported TTR in classes. The INR in these studies was ≥67% of time in therapeutic range in 72.0% of the patients.

**Conclusion:**

Reported quality of VKA treatment is highly dependent on the time-period since the start of treatment, with TTR ranging from approximately 56% in studies including the 1^st^ month to 75% in studies excluding the first 3 months.

## Introduction

Traditionally, patients with venous thromboembolism (VTE) are treated with low molecular weight heparins (LMWH) and vitamin K antagonists (VKA) such as warfarin, acenocoumarol or phenprocoumon [1], [2]. As with any medical treatment, the weighing of risks and benefits must be carefully balanced. The effect of VKA therapy depends on many factors including variation in dose response between patients, individual variation in pharmacokinetics and pharmacodynamic response, multiple interactions with food, co- medication and finally also by variation in adherence [3], [4]. VKA have a narrow therapeutic index, which needs to be monitored carefully in order to reduce the risk of tromboembolic events as well as bleeding complications [5]. With the large scale clinical testing of novel, direct acting oral anticoagulants, including the thrombin and factor Xa inhibitors dabigatran and rivaroxaban, a new era has been heralded. The main advantage of these new anticoagulants is the lack of a need for laboratory monitoring and dose adjustment due to more stable pharmacokinetics [6]. Several recent large randomized controlled trials have shown non-inferiority in effectiveness and safety of the new anticoagulants compared to VKA treatment [7], [8], [9], [10], [11]. However, the percentage of time within therapeutic range in the VKA-group, representing the quality of the control group, appears to vary considerably among these studies.

**Figure 1:**
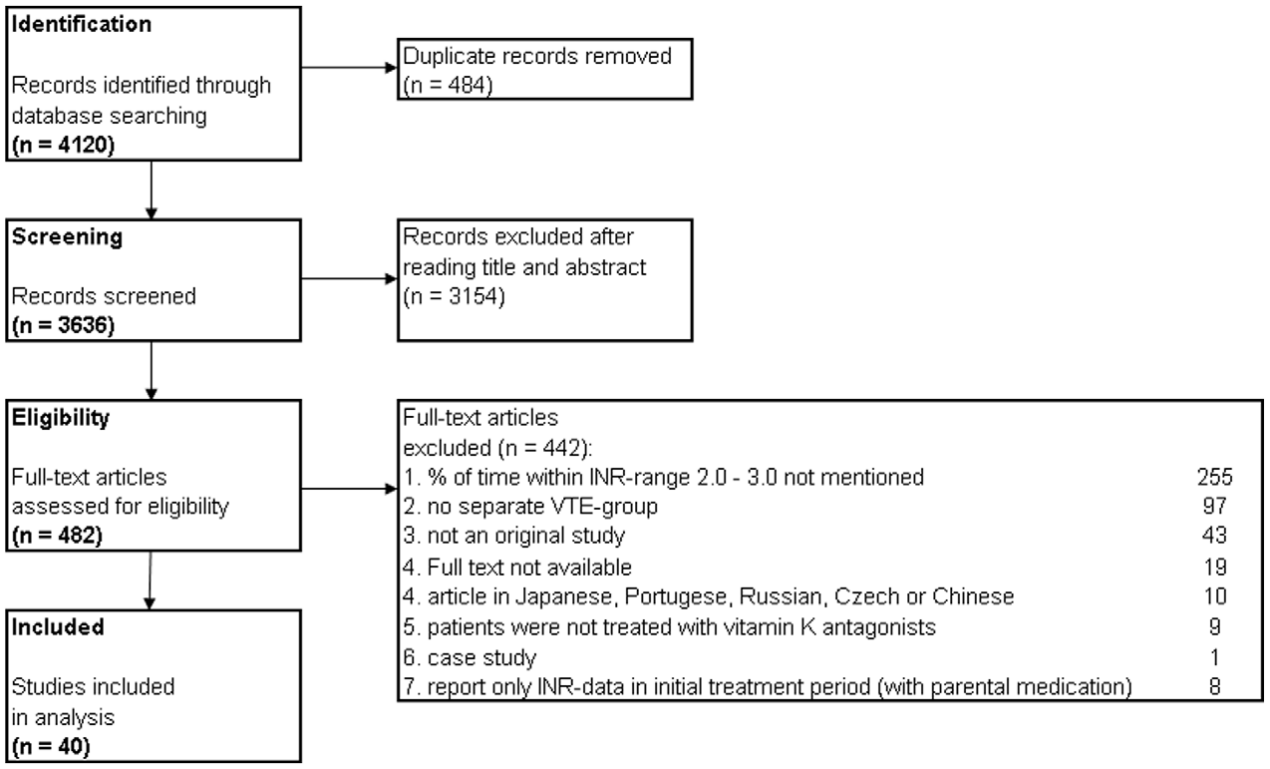
Flow diagram of literature search.

The International Normalized Ratio (INR), the ratio of a patient's prothrombin time to a normal (control) sample, raised to the power of the International Sensitivity Index (ISI) value, is established by the World Health Organization (WHO) and the International Committee on Thrombosis and Hemostasis for monitoring the effects of VKA. A target INR range of 2.0 to 3.0 is recommended for the treatment of VTE [3]. The most recognized way to measure the therapeutic effectiveness of VKA over time is to measure the percentage of time in the therapeutic range (TTR). TTR has been shown to strongly correlate with the clinical outcomes of hemorrhage or thrombosis and, thus, TTR is a reliable measure of the quality of anticoagulation management [12].

**Table 1:** Study characteristics of included studies.

	Study design/		% with	Included		Definition	Method
First author and year:	Type of study:	N:	cancer:	patients:	Treatment:	TR	measurement TTR:
Research Committee of		358	3.6%		UFH and Warfarin (4 weeks treatment)		
British Thoracic Society, 1992	RCT/duration OAC	354	2.8%	VTE	UFH and Warfarin (3 months treatment)	INR 2.0–3.0	Classes of %TTR
Agnelli, 2001	RCT/duration OAC	267	0%	DVT	LMWH and Warfarin/Acenocoumarol	INR 2.0–3.0	Linear Interpolation
Agnelli, 2003	RCT/duration OAC	326	0%	PE	LMWH and Warfarin/Acenocoumarol	INR 2.0–3.0	Linear Interpolation
Agnelli, 2007	RCT/dose-finding	126	<3%	DVT	LMWH and Warfarin/Acenocoumarol	INR 2.0–3.0	NR
		172	1%		LMWH and VKA local practice (outpatient treatment)		
Aujeskey, 2011	RCT/outpatients vs. inpatients	172	2%	PE	LMWH and VKA local practice (inpatient treatment)	INR 2.0–3.0	Linear Interpolation
Van Bladel, 2010	Cohort of PE patients	86	14%	PE	LMWH and Acenocoumarol	INR 2.0–3.5	Linear Interpolation
		104	100%				
Bona, 2000	Cohort with and without cancer	208	0%	VTE	Warfarin	INR 2.0–3.0	Linear Interpolation
		1103	10.2%		Fondaparinux and VKA local practice		
Büller, 2003	RCT/medication trial	1110	11.5%	PE	UFH and VKA local practice	INR 2.0–3.0	Linear Interpolation
					LMWH/UFH and Warfarin/Aceno-		
Büller, 2008	RCT/dose-finding	137	7.3%	DVT	coumarol/Phenprocoum/Fluindione	INR 2.0–3.0	Linear Interpolation
Caprini, 1999	Cohort/INR and DVT resolution	33	22.7%	DVT	UFH and Warfarin	INR 2.0–3.0	NR
Cassiopea Investigators, 2012	RCT/medication trial	1603	6%	PE	LMWH and Warfarin	INR 2.0–3.0	Linear Interpolation
		510	23.3%		LMWH and coumarin derivative		
Columbus Invesigators, 1997	RCT/medication trial	511	22.1%	VTE	UFH and coumarin derivative	INR 2.0–3.0	NR
Das, 1996	RCT/medication trial	55	5.5%	DVT	LMWH and Warfarin	INR 2.0–3.0	NR
Daskapoulos, 2005	RCT/medication trial	52	15.4%	DVT	UFH and Acenocoumarol	INR 2.0–3.0	NR
							after discontinuation LMWH
							until end of treatment,
Einstein Investigators, 2010	RCT/medication trial	1718	5.2%	DVT	LMWH and Warfarin/Acenocoumarol	INR 2.0–3.0	including interruptions
							after discontinuation LMWH
							until end of treatment,
Einstein Investigators, 2012	RCT/medication trial	2413	4.5%	PE	LMWH and Warfarin/Acenocoumarol	INR 2.0–3.0	corrected for interruptions
Fiesinger, 2005	RCT/medication trial	1249	13.5%	VTE	LMWH (Enoxaparin) and Warfarin	INR 2.0–3.0	NR
		360	22.2%		UFH and Warfarin		
Galilei Investigators, 2004	RCT/medication trial	360	21.1%	VTE	LMWH and Warfarin	INR 2.0–3.0	Classes of %TTR
		1452	9.5%	DVT			
Van Gogh Investigators, 2007	RCT/medication trial	1120	8.4%	PE	LMWH/UFH and Warfarin/Acenonocoumarol	INR 2.0–3.0	Linear Interpolation
Gonzalez-Fajardo, 1999	RCT/medication trial	80	10%	DVT	UFH and Coumarin	INR 2.0–3.0	Linear Interpolation
Heidinger, 2000	Cohort/self-management OAC	622	NR	VTE	VKA according to local practice	INR 2.0–3.0	Last 12 values
Kearon, 1999	RCT/duration OAC	162	0%	VTE	LMWH/UFH and Warfarin	INR 2.0–3.0	Linear Interpolation
Kearon, 2003	RCT/dose-finding	369	0%	VTE	LMWH/UFH and Warfarin	INR 2.0–3.0	Linear Interpolation
Kearon, 2004	RCT/duration of OAC	165	0%	VTE	LMWH/UFH and Warfarin	INR 2.0–3.0	Linear Interpolation
		355	16.6%		UFH and Warfarin		
Kearon, 2006	RCT/medication trial	353	15.0%	VTE	LMWH and Warfarin	INR 2.0–3.0	Linear Interpolation
		198	18.2%		UFH and VKA local practice		
Koopman, 1996	RCT/medication trial	202	16.8%	DVT	LMWH and VKA local practice	INR 2.0–3.0	Linear Interpolation
Levine, 1995	RCT/duration OAC	109	21.1%	DVT	UFH and Warfarin	INR 2.0–3.0	NR
López-Beret, 2001	RCT/medication trial	77	23.3%	DVT	LMWH (Nadroparin) and Acenocoumarol	INR 2.0–3.0	Linear Interpolation
Meyer, 2002	RCT/medication trial	75	100%	VTE	LMWH and Warfarin	INR 2.0–3.0	NR
Monreal, 1998	Cohort of VTE patients	244	13.5%	VTE	LMWH (Dalteparin) and Coumarin	INR 2.0–3.0	Linear Interpolation
Nielsen, 1993	RCT/medication trial	46	8.7%	DVT	UFH/Phenprocouman	INR 2.0–3.0	NR
		733	0%				
Palareti, 2000	Cohort with and without cancer	95	100%	VTE	Warfarin/Acenocoumarol	INR 2.0–3.0	Linear Interpolation
Pérez-de-Llano, 2010	RCT/medication trial	50	6%	PE	LMWH (Tinzaparin) and Acenocoumarol	INR 2.0–3.0	NR
Pini, 1994	RCT/medication trial	94	24.5%	DVT	Heparin and Warfarin	INR 2.0–3.0	Classes of %TTR
	RCT computer-assisted OAC	1560	NR		VKA local practice (Manual dosage)		
Poller, 2008	dosage vs medical staff	1649	NR	VTE	VKA local practice (Computer-assisted dosage)	INR 2.0–3.0	Linear Interpolation
Poli, 2007	Cohort of VTE patients	182	0%	VTE	LMWH/UFH and Warfarin	INR 2.0–3.0	Median % of time
		90	16.7%		LMWH/UFH and VKA local practice (stockings)		
Prandoni, 2004	RCT/intervention stockings	90	11.1%	DVT	LMWH/UFH and VKA local practice (no stockings)	INR 2.0–3.0	Classes of %TTR
Santamaria, 2006	Cohort/cost-effectiveness	116	17.2%	VTE	Acenocoumarol/Warfarin	INR 2.0–3.0	NR
							Classes of %TTR
Schulman, 1994	RCT/duration of OAC	1124	0%	VTE	LMWH/UFH and Warfarin	INR 2.0–2.85	Linear Interpolation
Schulman, 2009	RCT/medication trial	1265	4.5%	VTE	LMWH/UFH and Warfarin	INR 2.0–3.0	NR

**Abbreviations:** RCT, Randomized Controlled Trial; OAC, oral anticoagulation; UFH, Unfractionated Heparin; LMWH, Low Molecular Weight Heparin; VTE, Venous ThromboEmbolism; DVT, Deep Vein Thrombosis; PE, Pulmonary Embolism; TR, Therapeutic Range; INR, International Normalized Ratio; NR, not reported.

Dabigatran and rivaroxaban have been recently approved in many countries including the USA, Canada and also in Europe. This development will cause major changes in thrombosis management in the near future. Cost-effectiveness studies and real life registries will be the next step in the implementation of new oral anticoagulants. In order to adequately compare all treatment options, including novel anticoagulants and VKA, and to interpret the relative efficacy and safety of these novel anticoagulants, it is important to properly assess the quality of anticoagulant control, i.e. TTR, in the VKA group. This systematic review tries to provide a benchmark of TTR in patients with VTE receiving VKA and discusses the pros and cons of various ways to calculate TTR. Finally, it emphasizes the need to standardize TTR reporting, thereby contributing to a meaningful comparison among treatment options in studies evaluating novel anticoagulants.

**Table 2:** Quality assessment of the included studies.

		Reasons for	Incomplete data	Efforts to address		
	Consecutive	exclusion	adequately	potential sources		% loss to
First author and year	patients?	reported?	addressed?	of bias?	Follow-up time	follow-up
Research committee of						
British Thoracic	Yes	Yes	Yes	Yes	12 months	8%
Society, 1992						
Agnelli, 2001	Yes	Yes	Yes	Yes	at least 2 years	0%
Agnelli, 2003	Yes	Yes	Yes	Yes	at least 1 year	0%
Agnelli, 2007	Yes	Yes	Yes	Yes	at least 4	0%
					months	
Aujeskey, 2011	Yes	Yes	Yes	Yes	3 months	0.2%
Van Bladel, 2010	Yes	Yes	Yes	Yes	3 months	1.2%
Bona, 2000	Yes	No	Unclear	Unclear	Unclear	Unclear
Büller, 2003	Yes	Yes	Yes	Yes	3 months	0.5%
Büller, 2008	Unclear	No	Yes	Yes	3 months	0.7%
Caprini, 1999	Yes	Yes	No	No	6 months	Unclear
Cassiopea, 2012	Yes	Yes	Yes	Yes	6–12 months	Unclear
Columbus Investigators, 1997	Yes	Yes	Yes	Yes	3 months	0%
Das, 1996	Yes	Yes	Yes	Yes	3 months	Unclear
Daskapoulos, 2005	Yes	Yes	Yes	Yes	12 months	0%
Einstein Investigators, 2010	Yes	Yes	Yes	Yes	3–12 months	1%
Einstein Investigators, 2012	Yes	Yes	Yes	Unclear	Unclear	0.4%
Fiesinger, 2005	Unclear	Yes	Yes	Yes	6 ½ months	1.1%
Galilei Investigators, 2004	Yes	Yes	Yes	Yes	3 months	0%
Van Gogh Investigators, 2007	Yes	Yes	Yes	Yes	3 months	0.8%
Gonzalez-Fajardo, 1999	Yes	Yes	Yes	Yes	12 months	1.6%
Heidinger, 2000	No	Yes	No	Unclear	Unclear	Unclear
Kearon, 1999	Yes	Yes	Yes	Yes	Average 10	Unclear
					months	
Kearon, 2003	Yes	Yes	Yes	Yes	Average 2.4	0.1%
					years	
Kearon, 2004	Yes	Yes	Yes	Yes	12 months	0%
Kearon, 2006	Yes	Yes	Yes	Yes	3 months	0%
Koopman, 1996	Yes	Yes	Yes	Yes	6 months	1%
Levine, 1995	Unclear	Yes	No	Yes	12 months	3.4%
López-Beret, 2001	Yes	Yes	Unclear	Unclear	12 months	Unclear
Meyer, 2002	Yes	Yes	Unclear	Unclear	6 months	Unclear
Monreal, 1998	Yes	Yes	Unclear	No	3–6 months	Unclear
Nielsen, 1993	Yes	No	Unclear	Yes	3 months	Unclear
Palareti, 2000	Yes	Yes	Yes	Yes	Average 10–11	Unclear
					months	
Pérez-de-Llano, 2010	Yes	Yes	Yes	Yes	6 months	6%
Pini, 1994	Yes	Yes	Yes	Yes	9 months	0%
Poller, 2008	Unclear	Yes	Unclear	Yes	Average 17	Unclear
					months	
Poli, 2007	Yes	Yes	Yes	Yes	At least 1 year	8.1%
					or until	
					recurrence	
Prandoni, 2004	Yes	Yes	Yes	Yes	5 years	1.7%
Santamaria, 2006	Unclear	Yes	No	No	Median	Unclear
					98 days	
Schulman, 1994	Unclear	Yes	Yes	Yes	At least 6	Unclear
					months	
Schulman, 2009	Unclear	Yes	Yes	Yes	6 months	0.5%

## Materials and Methods

### Data sources and searches

A systematic search was performed to identify randomized controlled trials and cohort studies reporting the TTR in patients treated with VKA for deep vein thrombosis (DVT) confirmed by a non-compressible venous segment on an ultrasound of the extremities, or pulmonary embolism (PE) confirmed by an arterial filling defect on Computed Tomographic Pulmonary Angiography (CTPA) or a high probability ventilation/perfusion (V/Q) scan, or both (VTE). We searched Medline and Embase for articles in English, French, German, Dutch, Polish, Swedish, Danish, Italian and Spanish. Since the World Health Organization introduced the INR in 1983 [13] and the first studies reporting TTR in VKA in patients with VTE were published in the nineties, we searched for publications between January 1990 and May 2012. See Appendix 1 for detailed information about the search strategy and key words.

**Figure 2:**
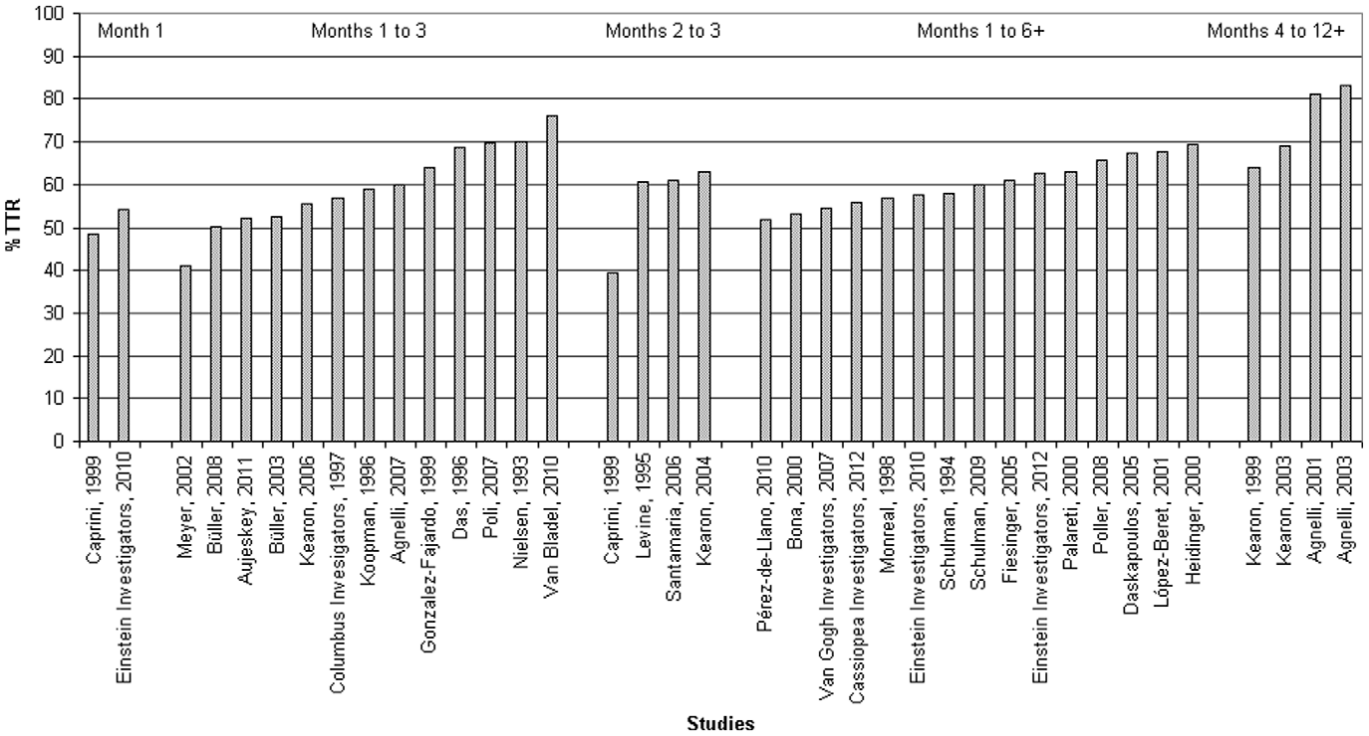
Time in Therapeutic Range in individual studies. * A weighted mean is calculated if a study reported more than 1 group; studies that only presented classes of %TTR are not represented here.

### Study selection

To be eligible for inclusion, studies had to fulfill the following criteria:

1. Study population consisted of consecutive adult patients with objectively confirmed DVT or PE.
2. Patients were treated with VKA for a minimum of three months.

Studies were excluded if they only reported the TTR in the initial treatment period while patients were still on parental medication such as low molecular weight heparin and unfractionated heparin.

**Table 3:** Percentage of time below, within and above the Therapeutic Range of the individual studies.

		Time-Period TTR		TTR	
First author and year:	N	since diagnosis:	% below TR	% within TR	% above TR
Research Committee of	358	Month 1	NR	37.4% (>67% TTR)	NR
The British Thoracic Society, 1992	354	Months 1 to 3	NR	35.2% (>67% TTR)	NR
Agnelli, 2001	267	Months 4 to 12+	NR	81%	NR
Agnelli, 2003	326	Months 4 to 12+	NR	83%	NR
Agnelli, 2007	126	Months 1 to 3	NR	60%	NR
Aujeskey, 2011	172		34.7%	51.8%	13.5%
	172	Months 1 to 3	32%	52.5%	15.6%
Van Bladel	86	Months 1 to 3	12.1%	76%	11.8%
	104		NR	47.5%	NR
Bona, 2000	208	Months 1 to 6+	NR	56%	NR
	1103		28%	53%	19%
Büller, 2003	1110	Months 1 to 3	28%	52%	20%
Büller, 2008	137	Months 1 to 3	29%	50.3%	20.7%
		At 1 month	42.4%	48.5%	12.1%
		At 3 monhts	63.6%	39.4%	3%
Caprini, 1999	33	At 6 months	48.5%	45.5%	9.1%
Cassiopea Investigators, 2012	1603	Months 1 to 6+	25.6%	55.9%	18.5%
	510		22%	57%	21%
The Columbus Invesigators, 1997	511	Months 1 to 3	24%	57%	21%
Das, 1996	55	Months 1 to 3	NR	68.8%	NR
Daskapoulos, 2005	52	Months 1 to 6+	19.1%	67.2%	13.6%
		Month 1	NR	54.1%	NR
The Einstein Investigators, 2010	1718	Months 1 to 6+	24.4%	57.7%	16.2%
The Einstein Investigators, 2012	2413	Months 1 to 6+	21.8%	62.7%	15.5%
Fiesinger, 2005	1249	Months 1 to 6+	NR	61%	NR
	360		NR	72.7% (≥70% TTR)	NR
The Galilei Investigators, 2004	360	Months 1 to 3	NR	70% (≥70% TTR)	NR
	1452		26.2%	54.4%	19.4%
The van Gogh Investigators, 2007	1120	Months 1 to 6+	26.9%	54.8%	18.3%
Gonzalez-Fajardo, 1999	80	Months 1 to 3	15%	64%	21%
Heidinger, 2000	622	on average at 4.5 months	22.7%	69.2%	8.1%
Kearon, 1999	162	Months 4 to 12+	22%	64%	14%
Kearon, 2003	369	Months 4 to 12+	20%	69%	11%
Kearon, 2004	165	Months 2 to 3	29%	63%	8%
	355		28%	55%	17%
Kearon, 2006	353	Months 1 to 3	25%	56%	19%
	198		18%	56%	26%
Koopman, 1996	202	Months 1 to 3	16%	62%	22%
Levine, 1995	109	Months 2 to 3	29.6%	60.7%	9.7%
López-Beret, 2001	77	Months 1 to 6+	22.8%	67.8%	9.4%
Meyer, 2002	75	Months 1 to 3	NR	41%	NR
Monreal, 1998	244	Months 1 to 6+	25.5%	56.9%	17.6%
Nielsen, 1993	46	Months 1 to 3	NR	70%	NR
	733		22.5%	63.6%	13.9%
Palareti, 2000	95	Months 1 to 6+	23.3%	58.9%	17.8%
Pérez-de-Llano, 2010	50	Months 1 to 6+	41.5%	51.7%	6.8%
Pini, 1994	94	Months 1 to 3+	NR	38% (≥67% TTR)	NR
	1560		NR	64.9%	NR
Poller, 2008	1649	Months 1 to 6+	NR	66%	NR
Poli, 2007	182	Months 1 to 3	18.8%	69.7%	11.5%
	90		NR	70% (>70% TTR)	NR
Prandoni, 2004	90	Months 1 to 3+	NR	72.2% (>70% TTR)	NR
Santamaria, 2006	116	At 3 months	NR	61.1%	NR
		At 12 months	NR	63% (≥75% TTR)	NR
Schulman, 1994	1124	Months 1 to 6+	25%	58%	17%
Schulman, 2009	1265	Months 1 to 6+	21%	60%	19%

**Abbreviations:** TTR, Time in Therapeutic Range; TR, Therpeutic Range; INR, International Normalized Ratio; NR, Not Reported.

### Data extraction and management

Three reviewers (PE, HTC, MP) operating in pairs of two extracted independently the following characteristics from each included study: study design, type of study (e.g. evaluation of a new drug, dose-finding, evaluation of duration of anticoagulation), characteristics of the study population (e.g. number of patients treated with VKA, country, inclusion criteria, proportion of patients with a malignancy), initial treatment, type of VKA (e.g. warfarin, acenocoumarol, phenprocoumon or other), initial dose of VKA, treatment duration, INR-monitoring by thrombosis service or self-management, percentage of time below therapeutic range (INR <2), percentage of time within therapeutic range (INR 2.0–3.0), percentage of time above therapeutic range (INR >3), method of calculation TTR and adverse events (e.g. recurrent VTE, major bleeding and mortality). The quality of the included studies was assessed by addressing the following issues: a) were consecutive patients included in the study?, b) did the authors report reasons for exclusion?, c) were incomplete data adequately addressed?, d) did the authors address potential sources of bias?, e) what was the duration of follow-up?, f) how many patients (percentage) were lost to follow-up?. Discrepancies were resolved by discussion. If agreement could not be reached a third reviewer was consulted.

**Table 4:** Weighted mean % of time below, within and above Therapeutic Range INR 2.0–3.0.

Time-period TTR			
INR 2.0–3.0	% below TR	% TTR	% above TR
since diagnosis	*Weighted mean*	*Weighted mean*	*Weighted mean*
**Month 1**			
(*n* studies = 2, *n* patients = 1751)	42.4%	54.0%	12.1%
**Months 1 to 3**			
(*n* studies = 13, *n* patients = 5473)	35.0%	55.6%	19.2%
**Months 2 to 3**			
(*n* studies = 4, *n* patients = 423)	32.9%	60.0%	8.1%
**Months 1 to 6+**			
(*n* studies = 13, *n* patients = 17338)	24.1%	60.0%	16.7%
**Months 4 to 12+**			
(*n* studies = 4, *n* patients = 1124)	20.6%	75.2%	11.9%

**Abbreviations:** TTR, Time in Therapeutic Range; INR, International Normalized Ratio; TR, Therapeutic Range.

### Data synthesis and analysis

A meta-analysis was performed by calculating a weighted mean, based on the number of participants in each included study, for each time-period in which the TTR was measured since the confirmation of the diagnosis of VTE.

**Figure 3:**
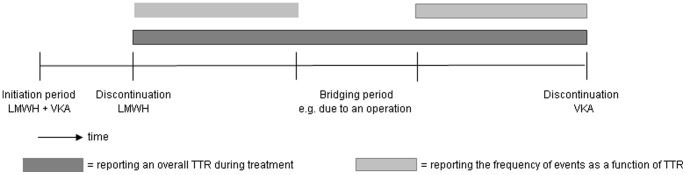
Suggestions for calculating TTR. Dark grey  =  reporting an overall TTR during treatment. Light grey  =  reporting the frequency of events as a function of TTR.

## Results

### Results of the search

The systematic search yielded 3636 citations. The results were screened and after reading titles and abstracts 3154 articles were excluded. Of the remaining 482 publications the full text was assessed. (Figure 1).

### Included studies

In total, 40 studies [8], [9], [14], [15], [16], [17], [18], [19], [20], [21], [22], [23], [24], [25], [26], [27], [28], [29], [30], [31], [32], [33], [34], [35], [36], [37], [38], [39], [40], [41], [42], [43], [44], [45], [46], [47], [48], [49], [50], [51] reporting the TTR in 26064 patients treated with VKA for VTE were included in the analyses.

Most studies included patients with DVT as well as PE. Fifteen studies [9], [14], [16], [19], [20], [21], [22], [25], [31], [32], [33], [36], [39], [42], [47] reported the results from patients with only DVT and eight studies [15], [18], [38], [47], [48], [49], [50], [51] reported the results from patients with only PE. The percentage of cancer ranged from 0% to 100%. The study characteristics of the included studies are presented in Table 1. The quality assessment of each study is shown in Table 2.

### Methods of calculating TTR

Five studies [24], [39], [42], [43], [45] reported TTR in classes ranging from <33% to ≥75% of time spent within INR-range 2.0 to 3.0. (e.g. 57% of all patients spent 70% of time within therapeutic range). All other TTRs were reported in percentages over time. Two studies [9], [20] reported the TTR in the first month since the start of treatment, thirteen studies [14], [18], [19], [21], [25], [29], [31], [34], [36], [40], [46], [48], [49] reported the TTR measured in months 1 to 3, four studies [20], [28], [32], [44] measured the TTR in months 2 to 3, fifteen studies [8], [9], [17], [22], [23], [26], [33], [35], [37], [38], [41], [45], [47], [50], [51] in months 1 to a minimum of 6 months and four studies [15], [16], [27], [30] reported the TTR in months 4 to at least 12 months since the start of treatment. Twenty (50%) studies [15], [16], [17], [18], [19], [25], [27], [28], [29], [30], [31], [33], [35], [37], [41], [45], [47], [48], [49], [50] reported that they calculated the TTR by using linear interpolation [52]. The method used for calculating TTR was not mentioned in 12 (30.0%) studies [8], [14], [20], [21], [22], [23], [32], [34], [36], [38], [44], [46] (Table 1).

### Percentage of time in therapeutic range

Table 3 presents the percentage of time below, within and above the therapeutic INR range of 2.0 to 3.0 of the individual studies. A histogram with the TTR in each individual study is given in Figure 2.

Table 4 details the weighted means for different time-periods since objective confirmation of the diagnosis VTE. The reported quality of VKA treatment is highly dependent on the time-period. In the first month the reported TTR is 54.0%. The TTR is 55.6% during the months 1 to 3 and 60.0% during a treatment of at least 6 months including the INRs in the first month. In studies reporting TTR without INRs in the first month, the TTR was 60.0% in months 2 to 3 since the start of treatment and 75.2% in the months 4 to 12 or longer.

## Discussion

A strong relationship between TTR and bleeding or thromboembolic rates has been observed across a large number of studies with different patient populations [53]. Since under-anticoagulation gives inadequate protection against thromboembolic events and over-anticoagulation increases the bleeding risk, it is important to report the quality of VKA treatment by using the TTR [54]. The evidence for non-inferiority of new anticoagulants depends on the quality of the VKA control group. The present review provides a benchmark of TTR in patients with VTE receiving VKA and discusses the pros and cons of various ways to calculate TTR.

We included 40 studies with more than 26000 participants and the results indicate that the achieved TTR ranges from approximately 56% to 75%.

The reported quality of VKA treatment was highly dependent on the time-period since the start of treatment. A statistically significant lower TTR was seen in studies reporting a TTR that covers all INRs, including the first month, compared to studies reporting the TTR without the first month. This difference is to be expected because of the difficulty to reach the therapeutic range in the initial treatment period and improvement in TTR during continuation of VKA treatment. Another explanation of the high TTR during longterm treatments is a selection-to-continue bias. Patients with stable INRs are more likely to continue their treatment with VKA than patients who experience problems in reaching the therapeutic range [55]. However, even after 4 to 12 months of treatment with VKA, patients spent 25% of their time outside of the therapeutic range.

Our review has some limitations that have to be mentioned. First, methods used to calculate TTR differed across the included studies. Fifty percent of the studies used linear interpolation, a few studies reported the percentage of time in a certain TTR class and 30% of the studies did not report the method of TTR calculation at all. Due to missing information about the exact calculation of TTR, we were unable to compare the different methods in a meaningful way. In literature, several methods to assess therapeutic control are described: e.g. the assessment of the number of INR measurements within the target range expressed as a percentage of the total number of INRs obtained, the cross-section-of the-files technique (the fraction of patients in range at one point in time compared to the total number of patients who had an INR at that point in time), equidivision, linear interpolation and the hybrid method [52], [56], [57]. Each approach has its advantages and disadvantages. A disadvantage of the first two methods is that they do not incorporate time and therefore cannot be used to calculate incidence rates of recurrences at different INR levels [54]. Time is incorporated in the method of equidivision, which assumes that the change between two consecutive INR measurements occurred halfway the interval [56]. The time spent in INR ranges can also be estimated by linear interpolation, which assumes that the INR between two measurements varies linearly from the first INR to the second INR [52]. A disadvantage of these last two methods is that extreme out of range INR values may bias overall results [58]. The hybrid method, in addition, takes effects of dosage modifications into account [54]. The results of all of these methods depend on whether an exact (INR 2.0–3.0) or an expanded therapeutic range is used, whether VKA-naïve patients (those just beginning therapy) are included or only patients already on established therapy, whether INRs obtained during invasive procedures when VKA therapy might be interrupted are excluded, and whether different oral anticoagulant preparations (e.g. warfarin or acenocoumarol) are allowed [53]. In a comparison of the equidivision, linear interpolation and hybrid methods, linear interpolation has been suggested as the preferred method as it shows a high validity and reproducibility [54]. We suggest that drug trials and real life registries with a VKA control group report the TTR in a uniform manner, to allow adequate comparison of data. Since linear interpolation has a high validity and was the most common method used to calculate TTR in the present review, we recommend to use linear interpolation in future studies covering the INRs from each patient from the discontinuation of heparin until the end of treatment. In order to avoid complex calculations, we believe that including time-periods with interruptions in VKA treatment in the TTR are acceptable. However, for calculating the relationship between TTR and adverse events, such as major bleeding episodes and thromboembolic events, we would suggest to exclude bridging periods, since the TTR will not represent the quality of anticoagulant treatment during these periods when most patients receive LMWH (Figure 3).

A second important limitation of the present review is that we were not able to investigate the association between TTR and clinical endpoints. Several studies in literature show a strong relationship between TTR and bleeding or thromboembolic events [53]. Unfortunately, data on such clinical endpoints related to TTR was not provided in the included studies.

Additionally, some other interesting sub-analyses were difficult due to small subgroups and the absence of detailed data. Hutten et al. indicated that the therapeutic quality of treatment was decreased when patients were treated with acenocoumarol rather than with warfarin [59]. This might implicate that the use of warfarin is preferable. However, since it is not clear whether these results might be influenced by factors such as frequency of monitoring and comorbidities, we need to be careful with drawing a conclusion. Furthermore, Hutten et al. showed that TTR was decreased in the presence of cancer and in the presence of a pulmonary embolism [59]. The same subgroup analyses in the present review did not show statistically significant results (data not shown). This might be explained by the fact that we did not have individual patient data (IPD). An IPD meta-analysis may give more detailed information for investigating such associations and may be interesting. Hutten et al. also showed a decrease in the therapeutic quality of VKA treatment when more than four changes in co-medication occurred [59]. Unfortunately such data was not available for our review.

The main conclusion of our systematic review is that the reported quality of VKA treatment is highly dependent on the time-period since the start of treatment, with the TTR ranging from approximately 56% in studies including the first month to 75% in studies excluding the first 3 months. The clinical consequences of our findings are not straightforward. However, it needs to be emphasized that the reported quality of VKA treatment should be taken into consideration while interpreting results from trials with new anticoagulants. Assuming an average treatment duration of 6 months, the mean TTR is approximately 60%. We recommend to calculate the TTR by using linear interpolation covering the INRs from each patient from discontinuation of heparin until the end of treatment. Furthermore, TTR is predictive of thromboembolic and bleeding complications for patients on VKA [53]; therefore a proper calculation of TTR in the VKA group is of importance in assessing the adequacy and quality of novel anticoagulants.

Oral anticoagulants are also effective in preventing stroke [60], [61], [62], [63], [64] and prolonging survival rates in patients with atrial fibrillation (AF) [65]. It may be interesting to investigate a benchmark of the TTR in patients treated with VKA in AF in the near future. However, since patients with AF are usually on long-term VKA treatment, selection-to-continue bias will be more evident than in patients with VTE and should be taken into consideration in an analysis in AF patients [55].
